# Tip110/SART3 regulates IL-8 expression and predicts the clinical outcomes in melanoma

**DOI:** 10.1186/s12943-018-0868-z

**Published:** 2018-08-17

**Authors:** Khalid Amine Timani, Balázs Győrffy, Ying Liu, Khalid S. Mohammad, Johnny J. He

**Affiliations:** 1 Department of Microbiology, Immunology, and Genetics, University of North Texas Health Science Center, 3500 Camp Bowie Blvd, Fort Worth, TX 76107 USA; 2 MTA TTK Lendület Cancer Biomarker Research Group, Institute of Enzymology, Magyar Tudósok körútja 2, Budapest, 1117 Hungary; 3 Semmekweis University 2nd Department of Pediatrics, Tűzoltó utca 7-9, Budapest, 1094 Hungary; 4 Division of Endocrinology, Department of Internal Medicine, Indiana University School of Medicine, Indianapolis, IN 46202 USA

**Keywords:** Tip110/SART3, IL-8, Melanoma, Hypoxia, TP53, NF-κB

## Abstract

Tip110, an important regulator of several oncogenic proteins, was significantly downregulated in human metastatic melanoma cells exposed to a hypoxic condition. Therefore, in this study, we set to determine whether differential expression of Tip110 could be an important indicator for melanoma tumorigenesis and metastasis. We found that in melanoma, but not in other cancer types, Tip110 knockdown enhanced significant expression and secretion of IL-8 and melanoma cells invasions. This induction was further potentiated under hypoxia and by inflammatory cytokine and found independent of TNF-α autocrine signaling. We further showed that Tip110 knockdown-mediated IL-8 induction involved IL-8 mRNA stability. Furthermore, the transcriptomic profiling data and survival from 455 melanoma patients demonstrated that the correlation between Tip110 expression and the clinical outcomes in melanoma was stage-dependent. These findings uncover important roles of Tip110 in melanoma tumorigenesis and metastasis through regulation of IL-8 and hope to provide new clues for future therapeutic strategies.

**Electronic supplementary material:**

The online version of this article (10.1186/s12943-018-0868-z) contains supplementary material, which is available to authorized users.

## Main text

Malignant melanoma is an aggressive form of skin cancer and one of the leading causes of death in the United States. The demonstration of an increased incidence of metastasis at sites of inflammation supports the important role of inflammatory cytokines in the pathogenesis of tumor growth and metastasis. Among these cytokines is interleukin-8 (IL-8), which is well documented for its important roles in melanoma tumorigenesis and metastasis [1]. IL-8 is normally induced and secreted by a diverse range of cells in response to pathologic stresses. Its serum levels in patients are significantly elevated and are correlated with advanced disease stage and poor prognosis. Thus, identification of the molecular players regulating IL-8, and in return promoting melanoma initiation and progression, will provide new clues for possible therapeutic strategies against melanoma.

HIV-1 Tat-interacting protein (Tip110), also known as squamous cell carcinoma antigen recognized by T cells 3 (SART3), is a multifaceted nuclear protein and has been shown to function in tumor antigenicity, regulation of gene transcription, mRNA synthesis, stem cell proliferation and differentiation, and embryogenesis [2]. We and others have reported that Tip110 interacts with and/or regulates several oncogenic proteins [2]. The high expression level of Tip110 has been found in a number of malignant tumor cell lines and cancerous tissues [2]. However, exposure of highly metastatic melanoma cells to hypoxia led to a significant downregulation of Tip110 together with TP53 in both in vitro and in a melanoma cancer bone metastasis mouse model [3]. Also, Tip110 expression was found to be dysregulated and correlated with a metastatic phenotype in melanoma and lung adenocarcinoma [4, 5]. Therefore, it is plausible that differential expression of Tip110 could be an indicator for melanoma tumorigenesis.

Here, we demonstrated that Tip110 contributed to the regulation of the expression of IL-8 in melanoma cells and that the expression level of Tip110 had a prognostic value for melanoma patients. Furthermore, we identified the potential mechanism underlying the Tip110 knockdown-induced IL-8 expression.

## Results and discussion

### Tip110 knockdown induced IL-8 expression in melanoma with and without stimuli

**Fig. 1:**
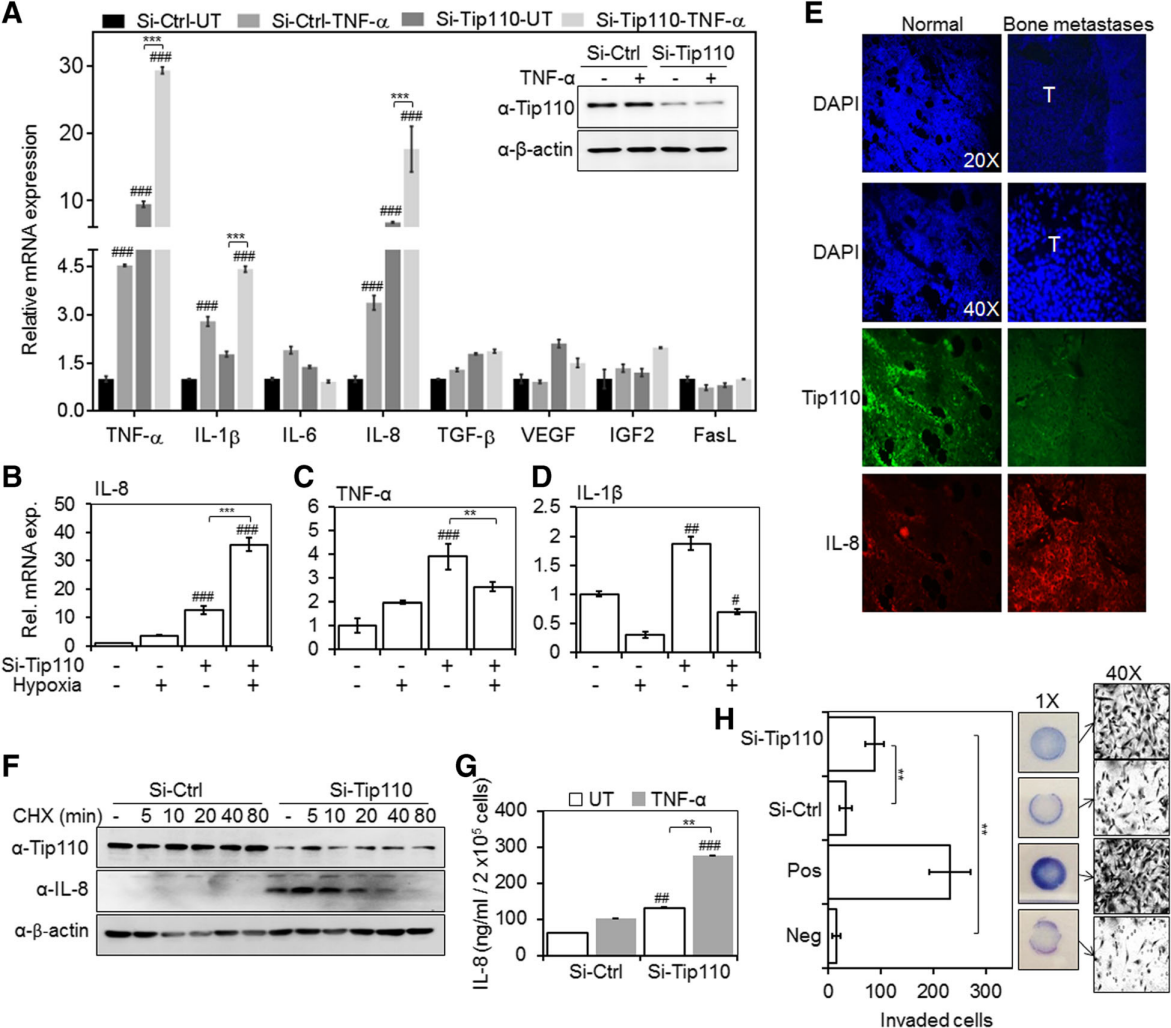
Negative correlation between Tip110, and IL-8 expression, secretion and melanoma cell invasion. **a**. Melanoma cancer cells 1205Lu were transfected with either si-Ctrl or si-Tip110, cultured for 48 h, treated with 10 ng/ml of TNF-α for 4 h, and harvested for total RNA isolation. qRT-PCR was performed to determine the mRNA expression of the indicated proteins. Knockdown of Tip110 protein was confirmed by Western blotting (inset panel). **b-d**. 1205Lu were transfected same as above, cultured under severe hypoxia (0.3% O_2_) for 48 h, and harvested for total RNA isolation and qRT-PCR to determine the (**b**) IL-8, (**c**) TNF-α and (**d**) IL-1β mRNA level. β-actin was included in the qRT-PCR and used as a relative reference. The mRNA levels in the cells transfected with si-Ctrl and untreated with TNF-α were set to 1. **a-d**. # *P < 0.05, ## P < 0.01*, ### *P < 0.001* compare to si-RNA untreated or normoxia control (si-Ctrl); ** *P < 0.01*, *** *P < 0.001*; 2-way ANOVA. **e** Mouse osteolytic bone metastasis tissues were obtained by inoculating 1205Lu into bones of athymic nude mice. Longitudinal sections of control or metastasis bone were prepared and immunostained for Tip110 and IL-8 protein expression. DAPI was used to visualize the nuclear DNA. T; tumor core region. **f**. 1205Lu were transfected with si-Tip110 or si-Ctrl, treated with cycloheximide (CHX) for 0, 5, 10, 20, 40 or 80 min, and harvested for cell lysates and Western blotting for Tip110 and IL-8 protein expression. β-actin was used as loading control. **g**. Cells were transfected and treated with TNF-α as stated above. Cell culture supernatants were collected 24 h post TNF-α treatment and analyzed for IL-8 using a human IL-8-specific ELISA kit. *## P < 0.01*, ### *P < 0.001*, compare to si-RNA untreated control (si-Ctrl); ** *P < 0.01*; 2-way ANOVA. **h**. si-Tip110 or si-Ctrl-transfected 1205Lu were seeded on a 24-well BioCoat™ Matrigel® invasion chamber with 8.0 μm PET membrane permeable supports. Media containing 0.1 and 10% FBS was used as the negative (Neg), and positive (Pos) controls, respectively. Conditioned media from si-Ctrl and si-Tip110 transfected cells contained 0.1% FBS were incubated for 16 h. Invaded cells were counted in 10 individual fields, and phase contrast images at 1× and 40× were captured. ** *P < 0.01*; Student *t*-test. These results represent three independent experiments and the data presented as the mean ± SE. UT; Untreated

Many alterations in tumor cells have been implicated as contributing factors to the tumor progression and metastasis. These changes include phenotypic characteristics such as autocrine expression of cytokines/growth factors and oncogenes or inactivation of tumor suppressor genes and loss of responsiveness to inhibitory cytokines/growth factors. Several reports had shown that human melanoma cells grown in cultures express and secrete numerous cytokines/growth factors either constitutively or upon induction [6]. In addition, our previous studies have shown a significant reduction of Tip110 together with TP53 expression in melanoma cells exposed to a hypoxic condition [3]. Therefore, we examined the expression of selected inflammatory cytokines and growth factors that are important during melanoma progression, metastasis, and survival, in the context of limited Tip110 expression [6]. Since TNF-α is involved in the biosynthesis of many other cytokines, we speculated that TNF-α treatment would potentiate any effect that could be exerted by Tip110 knockdown. The results showed that Tip110 knockdown led to significant up-regulation of both IL-8 and TNF-α mRNA with and without TNF-α treatment (Fig. 1a). A moderate increase was found with IL-1β**.** Furthermore, the kinetic and the level of IL-8 mRNA expression pattern with Tip110 knockdown and TNF-α treatment was different from TNF-α and IL-1β expression at different time points (Additional file 1: Figure S1A-C) indicating that IL-8 induction by Tip110 knockdown in melanoma cells is more likely independent of autocrine TNF-α and IL-1β regulations. Induction of IL-8 by Tip110 knockdown was also observed in other tested melanoma cell lines (Additional file 1: Figure S2A-D), while no such effect was observed in the cell lines from other cancer types (Additional file 1: Figure S2E-I) except for non-small-cell lung cancer cells, H1299, but the magnitude of IL-8 induction was relatively small compared to that in melanoma cells with TNF-α treatment (Additional file 1: Figure S2J).

IL-8 mRNA is highly expressed in hypoxic areas of several tumors including malignant melanoma, glioblastoma and ovarian carcinoma [1]. Thus, we examined the expression level of selected cytokines in response to a severe hypoxic condition and Tip110 knockdown. qRT-PCR analysis revealed that hypoxia-induced up-regulation of IL-8 was significantly enhanced by Tip110 knockdown in melanoma (Fig. 1b). Unlike IL-8, the expression of TNF-α and IL-1β in response to hypoxia and Tip110 knockdown was reduced (Fig. 1c and d). In addition, Tip110 expression was suppressed in the core tumor and presumably severe hypoxic area of mouse bone metastasis tissues obtained by inoculating metastatic melanoma 1205Lu ([3] and Fig. 1e).

Tip110 knockdown in 1205Lu cells increased IL-8 protein expression and stability compared to control transfected cells by using cycloheximide (CHX) chase assay (Fig. 1f). Using ELISA assays, we also detected increased levels of IL-8 in the conditioned media from Tip110-knockdown 1205Lu cells with and without TNF-α treatment (Fig. 1g**)**. Furthermore, conditioned media of Tip110-knockdown cells significantly increased the invasiveness of melanoma cells (Fig. 1h). These results suggest that Tip110 downregulation in melanoma cells leads to enhanced cell invasion, likely through induction of IL-8 production and release as a growing number of studies have shown that IL-8 is associated with tumor cells migration and invasion [1].

### Tip110 regulated IL-8 mRNA stability

**Fig. 2:**
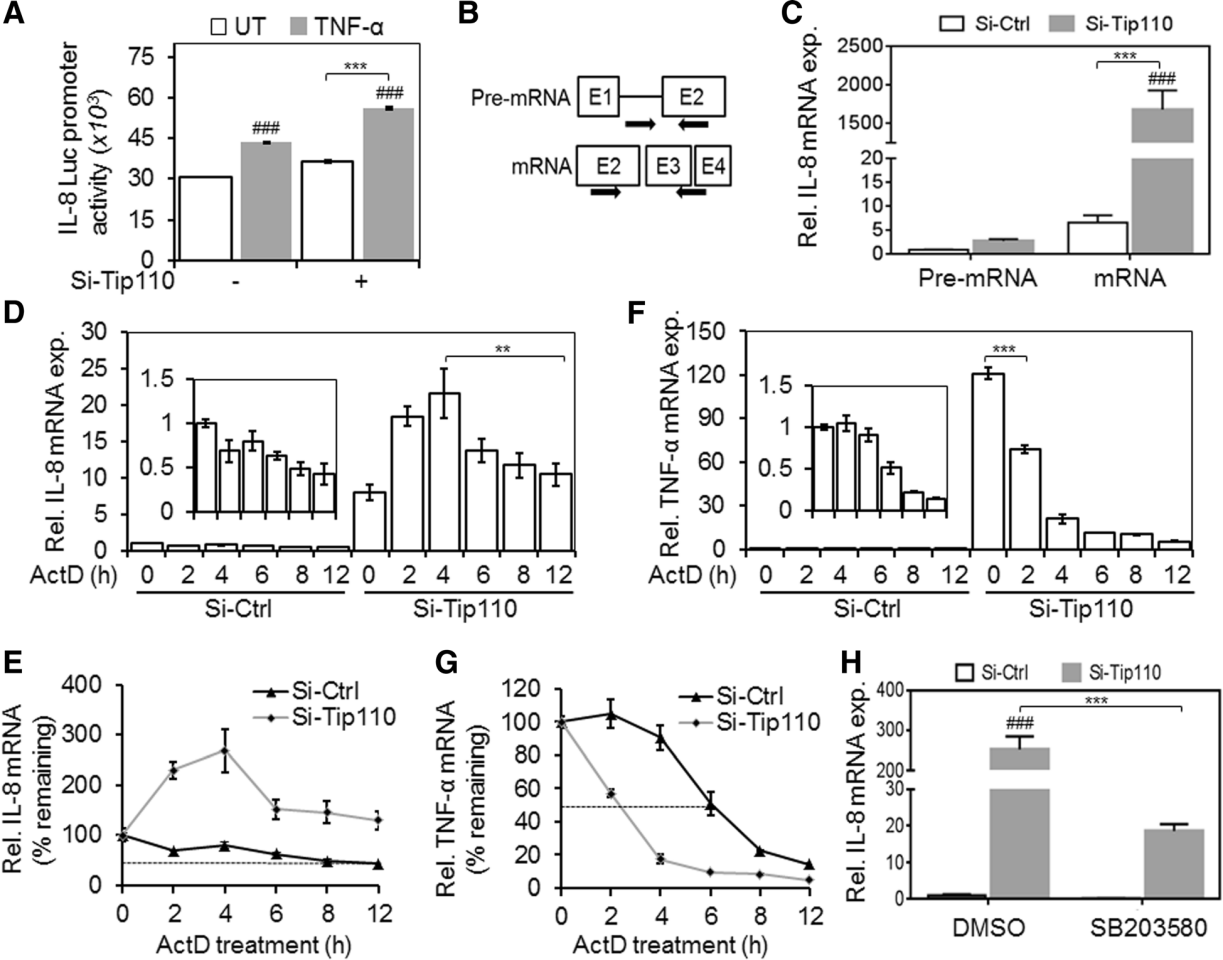
Effects of Tip110 knockdown on the IL-8 mRNA stability. **a**. 1205Lu were transfected with pIL-8.Luc.pro reporter vector plus si-Tip110 and si-Ctrl and cultured for 48 h. The cells were treated with 10 ng/ml TNF-α for 20 h. pTK-βgal was used to normalize the transfection efficiency variations among all transfections. ### *P < 0.001* compare to si-RNA untreated control. *** *P < 0.001*; 2-way ANOVA. **b**. Schematic for the primer sets on the *IL-8* (indicated by arrow) used for qRT-PCR to determine the levels of IL-8 pre-mRNA and mRNA. **c.** 1205Lu were transfected with si-Tip110 or si-Ctrl, cultured for 48 h, and harvested for total RNA isolation and qRT-PCR to determine the level of IL-8 pre-mRNA and mRNA. The pre-mRNA levels in si-Ctrl-transfected cells were set to 1. ### *P < 0.001*, compare to si-Ctrl pre-mRNA, *** *P < 0.001*; 2-way ANOVA. **d-g**. 1205Lu were transfected same as above, treated with 5 μg/ml actinomycin D (ActD) for 0, 2, 3, 6, 8, or 12 h, and harvested for RNA isolation and qRT-PCR to determine the mRNA levels of IL-8 (**d**) and TNF-α (**f**). β-actin and GAPDH were included in the qRT-PCR and used as a relative reference. The mRNA levels of si-Ctrl-transfected cells at 0-time point were set to 1. ** *P < 0.01*, *** *P < 0.001*; Student *t*-test. The percentage of the remaining IL-8 mRNA (**e**) and TNF-α mRNA (**g**) were calculated by setting their respective mRNA level of the transfected cells at 0 h to be 100. The dashed lines indicated the 50% mRNA half-life. **h**. 1205Lu were transfected as above, and treated with 5 μM p38 MAPK inhibitor SB203580, or the vehicle control DMSO for 5 h, and harvested for RNA isolation and qRT-PCR for the IL-8 mRNA level. ### *P < 0.001*, compare to si-Ctrl control, *** *P < 0.001*; 2-way ANOVA. All the data are representative of triplicate independent experiments and the data presented as the mean ± SE. UT, Untreated

The regulation of IL-8 expression involves both transcriptional and posttranscriptional mechanisms [7]. Tip110 knockdown showed modest activation of the IL-8 promoter activity (Fig. 2a) indicating that induction of IL-8 by Tip110 knockdown does not solely rely on the IL-8 transcriptional activity. IL-8 could also potentially be induced in cancer cells at the level of RNA synthesis and/or mRNA stability [7]. Considering the role of Tip110 in RNA processing [2], we investigated whether Tip110 knockdown would induce IL-8 through affecting the processing of *IL-8* gene transcripts and their stability. Using specific primer sets **(**Fig. 2b**),** which specifically distinguish IL-8 pre-mRNA and mature mRNA, we found that in contrast to increases of the IL-8 pre-mRNA, the IL-8 mature mRNA was disproportionally increased in si-Tip110-transfected cells (Fig. 2c). These results indicate that Tip110 likely regulates IL-8 mRNA stability rather than IL-8 pre-mRNA synthesis. To further address this possibility, we performed an actinomycin D (ActD) chase assay. The results showed that Tip110 knockdown significantly prolonged half-life of the IL-8 mRNA compared to the control (Fig. 2d and e). Previously, we showed that Tip110 knockdown up-regulated TNF-α mRNA (Fig. 1). Therefore, we examined whether Tip110 would affect TNF-α mRNA stability in a similar fashion to the IL-8. Interestingly, TNF-α mRNA was highly unstable by knockdown Tip110 (Fig. 2f and g), although the initial mRNA level at 0-h time point was much higher.

Since the p38 MAPK signaling pathway is known to play a major role in IL-8 post-transcriptional regulation and mRNA stability [7], we determined the roles of Tip110 knockdown regulation of IL-8 transcript levels. The results showed that treatment of cells with p38 MAPK signaling inhibitor led to significant attenuation of IL-8-up-regulation at the mRNA level in Tip110 knocked down cells (Fig. 2h). These results demonstrated that Tip110 knockdown specifically stabilized and sustained the *IL-8* gene transcripts, most likely through p38 MAPK signaling.

Tip110 has the ability to recognize and bind to RNA, and early studies have revealed that Tip110 plays a role in the regulation of mRNA synthesis [2]. However, Tip110 knockout in zebrafish can trigger compensatory responses by up-regulating other mRNA synthesis related components [8]. Unexpectedly, in Tip110 knockdown cells, we found an increase in IL-8 mRNA after short-time exposure to ActD (Fig. 2d and e). We speculate that the unexpected findings may be due to robust and high kinetics rate of IL-8 mRNA synthesis compared to TNF-α mRNA in the context of a limited amount of Tip110. Indeed, It has been shown in primary human monocytes and macrophages that there is a much stronger and sustained novel type of IL-8 mRNA stabilization compared to TNF-α mRNA [9]. The (AU)-rich sequence in the 3’-UTR of the IL-8 gene has been suggested to contribute to its post-transcriptional regulation [10]. Therefore, it is possible that Tip110 targets the 3’-UTR of IL-8 and regulates its mRNA stability in cancer.

### Stage-dependent expression of Tip110 correlated with the clinical outcomes of melanoma patients

**Fig. 3:**
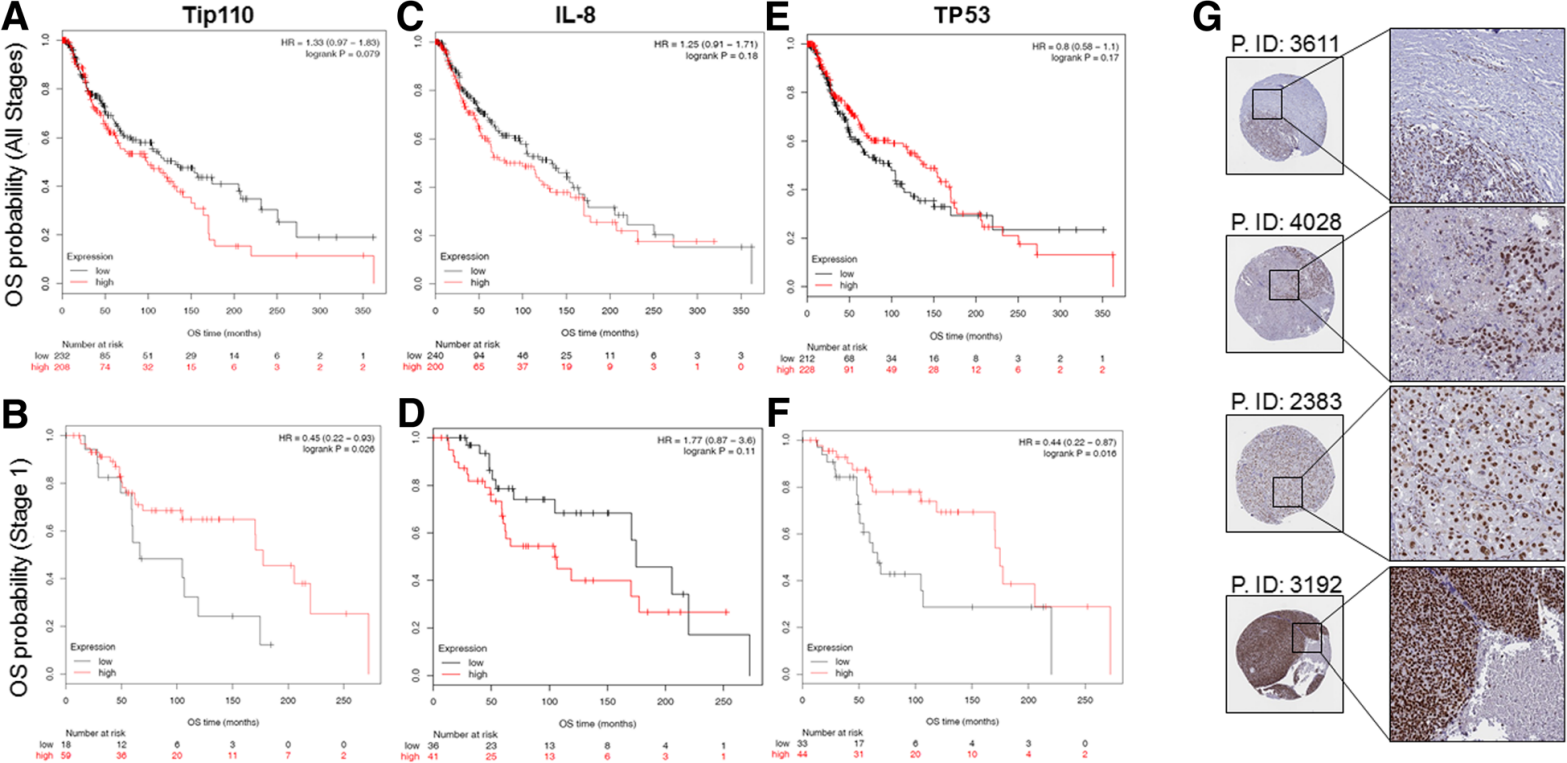
Correlation between Tip110, IL-8, TP53 expression and clinical outcome in melanoma patients. RNA-Seq data from TCGA were used to assess the correlation between Tip110 expression and the overall survival (OS) probability (**a** and **b**), between IL-8 expression and the OS probability (**c** and **d**), and between p53 expression and the OS probability (**e** and **f**). The OS probability was calculated separately for all stages melanoma patients (**a**, **c** and **e**) and for stage 1 melanoma patients (**b**, **d** and **f**), which is defined as melanoma that is up to 2 mm thickness (Additional file 1: Figure S3E). HR, hazard ratio. **g**. Tip110 expression in malignant melanoma cancer tissues. Images credit: Human Protein Atlas, (www.proteinatlas.org) [13]. Images are available at the following URL: v16.proteinatlas.org/humancell. P.ID: patient identification number

To determine whether Tip110 expression would be correlated with clinical outcomes of melanoma patients, we mined the transcriptomic profiling data and survival of melanoma patients available in the TCGA database. The median follow-up was 28.8 months in the analyzed cohort that was composed of 455 (female = 180 and male = 289) adult melanoma cancer patients (mean age: 58 years). Of all patients, 417 had no evidence of metastasis (M = 0) while 24 patients had melanoma spread from the primary site to other areas of the skin or under the skin, or distant lymph nodes (M = 1) **(**Additional file 1: Figure S3A). The patients were further grouped based on the melanoma stage, primary tumors (T) and regional lymph nodes (N) (Additional file 1: Figure S3C-F). The survival analysis using all patients in the database showed that melanoma cancer patients with high Tip110 expression experienced worse overall survival rates (Fig. 3a). Interestingly, when we ran the survival analysis using patients at “stage 1” (*n* = 77); which was defined as melanoma that is up to 2 mm thick, no lymph node involvement and no distal metastasis; the patients with low Tip110 expression experienced significantly worse overall survival rates (Fig. 3b). We further analyzed the overall survival of melanoma patients with respect to IL-8 expression and found no significant difference in the overall survival of either all patients or the patients with “stage 1” tumors (Fig. 3c and d). However, there was an inverse correlation on the overall survival between the Tip110 and IL-8 expression at the “stage 1”. Recently, it has been reported that changes in serum IL-8 levels during the immunotherapy treatment correlate with overall survival in patients with metastatic melanoma and non-small-cell lung cancer [11].

The results further demonstrated that TP53 expression, which is found to be regulated by Tip110 [3], at “all stages” was not prognostic in malignant melanoma, while at “stage 1” low expression of p53 showed poor prognosis similar to the Tip110 in the same patient subpopulation (Fig. 3e and f). When we divided the samples into two cohorts according to the TP53 status, we found no significant correlation between TP53 status and Tip110 expression (data not shown). 89% of all melanoma patients (*n* = 387) in our study expressed wild-type TP53 (Additional file 1: Figure S3B). An early study has shown that an increased level of TP53 protein does not indicate an increased degree of malignancy in melanoma, but rather suggests a more favorable disease progression [12]. In addition, TP53 is also tightly regulated at the translational level through phosphorylation and ubiquitination while the overall survival data were obtained based on the TP53 mRNA level.

When we examined the Tip110 expression in melanoma patients’ tissues using the human protein atlas online tool [13], we found several melanoma tumor samples show a high level of Tip110 expression. Interestingly, some patients’ samples appeared to have a high degree of heterogeneity in Tip110 expression throughout a tissue section (Fig. 3g, subset). The overall findings suggest the importance of differential expression of Tip110 in melanoma tumorigenesis and its potential prognostic value in patients’ clinical outcomes.

## Conclusions

Our findings demonstrated the importance of the differential expression of Tip110 as a prognostic indicator of melanoma tumorigenesis and the potential link to tumor metastasis. Moreover, the roles of Tip110 in regulating the production of IL-8 in melanoma suggest that the target genes or regulators of Tip110 may be suitable for therapeutic intervention.

## Additional files

Additional file 1: Supplementary materials. (ZIP 3620 kb)

## Abbreviations

ActD: Actinomycin D
CHX: Cycloheximide
ELISA: Enzyme-linked immunosorbent assay
Fas L: Fas ligand
IGF2: Insulin-like growth factor 2
IL1β: Interleukin 1 beta
IL-6: Interleukin 6
IL-8: Interleukin 8
p38 MAPK: p38 mitogen-activated protein kinases
SART3: Squamous cell carcinoma antigen recognized by T cells 3
TCGA: The cancer genome atlas
TGF-β1: Transforming growth factor beta 1
Tip110: HIV-1 Tat-interaction protein
TNF-α: Tumor necrosis factor-alpha
TP53: Tumor protein 53
VEGF: Vascular endothelial growth factor
