# Supplementary material for: Tip110/SART3 regulates IL-8 expression and predicts the clinical outcomes in melanoma
Source: Mol Cancer. 2018 Aug 17;17:124. doi: 10.1186/s12943-018-0868-z (PMC6098614; doi:10.1186/s12943-018-0868-z)
Supplement: Supplementary file 1 — Supplementary materials. (ZIP 3620 kb) [file 12943_2018_868_MOESM1_ESM.zip › List of Primers.pdf]

Table –S1 List of primers used in this study for the real-time PCR

Gene	Forward primer (5′ to 3′)	Reverse primer (5′ to 3′)
SART3	GGCTAGGAT TGAGGCTCG ACTG	GGGTGTCACCATGAGCTCTTTCC
TNF-α	TCTTCTCGAACCCCGAGTGA	CCTCTGATGGCACCACCAG
IL-8	TGCCAAGGAGTGCTAAAG-3′	TCTCAGCCCTCTTCAAAA
IL-6	ACAACAAATTCGGTAGATCCTCG	AGCCATCTTTGGAAGGTTCAGG
IL-1α	CGCCAATGACTCAGAGGAAGA	AGGGCGTCATTCAGGATGAA
Il-1β	CCTGTCCTGCGTGTTGAAAGA	GGGAACTGGGCAGACTCAAA
TGF-β	CGTGGAGCTGTACCAGAAATAC	ACAACTCCGGTGACATCAAA
VEGF	AGGGCAGAATCATCACGAAG	CACACAGGATGGCTTGAAGA
IGF2	GTTCGGTTTGCGACACG	AGAAGCACCAGCATCGACTT
FasL	GGATTGGGCCTGGGGATGTTTCA	TTGTGGCTCAGGGGCAGGTTGTTG
β-actin	AAACTGGAACGGTGAAGGTG	AGAGAAGTGGGGTGGCTTTT
GAPDH	GAAGGTGAAGGTCGGAGTCA	TTGAGGTCAATGAAGGGGTC
