# Supplementary material for: Tip110/SART3 regulates IL-8 expression and predicts the clinical outcomes in melanoma
Source: Mol Cancer. 2018 Aug 17;17:124. doi: 10.1186/s12943-018-0868-z (PMC6098614; doi:10.1186/s12943-018-0868-z)
Supplement: Supplementary file 1 — Supplementary materials. (ZIP 3620 kb) [file 12943_2018_868_MOESM1_ESM.zip › Materials and Methods plus Supp Figures S1-S3.pdf]

Materials and methods

Cell culture and transfection

The parental metastatic melanoma cell line 1205Lu (22) was grown in a composite medium (W485) consisting of 3 parts MCBD153 medium (Sigma-Aldrich, St. Louis, MO) and 1 part L15 medium (Sigma-Aldrich), supplemented with 4 % fetal bovine serum (FBS) and 2 mM L-glutamine (Sigma-Aldrich). SK-Mel-2, SK-Mel-5, Malme-3M, HEK 293T, U2OS, Saos-2 (ATCC, Manassas, VA), human foreskin fibroblasts (HFF; a gift from Dr. Ann Roman, Indiana University School of Medicine) were cultured in Dulbecco's modified Eagle's medium (DMEM; Sigma-Aldrich). COLO829, H1299 and SW480 cells (ATCC) were cultured in RPMI-1640 medium (Sigma-Aldrich). SH-SY5Y cell line was grown in 1:1 mixture of DMEM:F12 media. 10 % FBS, 100 IU/ml penicillin, and 100 µg/ml streptomycin (Gibco) were added to all media. Plasmid DNA transfection was performed using the standard calcium phosphate precipitation method for HEK 293T. All other cell lines were transfected by using Lipofectamine 2000 (Invitrogen life technologies, Grand Island, NY) according to the manufacturer's instructions for both DNA and siRNA.

DNA plasmids and siRNAs

The construction of Tip110.His plasmid was described elsewhere (23). The promoter fragment (-1400 to -1) upstream of a human IL-8 gene was cloned from human embryonic stem cells by standard PCR techniques and then inserted in front of the

luciferase gene in the reporter plasmid pGL3-basic (Promega) using the following primers: 5'-ATA GGG TAC CCG TCA TAC TCC GTA TTT G-3' and 5'-ATA GCT CGA GCA AAT ACG GAG TAT GAC G-3'. Respective cloning sites KpnI and XhoI were introduced in the primers as underlined. The correct sequence of the clone was confirmed by restriction digestion followed by sequencing. ON-TARGET plus (SMART pool) Tip110 siRNA (L-013447-01) and control On-TARGET plus Non-targeting pool siRNA (D-001810-10) were designed and purchased from Dharmacon. p53 siRNA was purchased from Sigma-Aldrich.

Western blotting

Cells were washed in cold PBS and lysed in the Whole cell lysates extract buffer (WCEB) (50 mM Tris.HCl, pH 8.0, 280 mM NaCl, 0.5 % NP-40, 0.2 mM EDTA, 2 mM EGTA, 10 % glycerol, 2 mM PMSF and protease inhibitor cocktail (Sigma-Aldrich)). Lysates were cleared of cell debris by centrifugation at 12,000 *xg* and fractionated on SDS-PAGE, followed by Western blotting analysis.

Immunohistochemistry

Paraffin-embedded sections on slides were deparaffinized. The tissue sections were placed in the target retrieval solution (Dako) and treated at 95 °C for 40 min. After cooling to the room temperature, the tissues were permeabilized in 0.2 % Triton X-100 for 10 min then washed with PBS. The tissues were blocked with 3 % BSA and

incubated with an anti-IL-8 (ab106350, Abcam) and anti-Tip110 (14) antibodies (1:500) for overnight at 4 C° in a humidified chamber. Then the tissues were washed and incubated with either anti-Alexa Fluor 555 or anti-Alexa Fluor 488 secondary antibodies (Invitrogen) (1:500) for 1 hr and then 1 ng/ml 4,6-diamidino-2-phenylindole (DAPI) for 10 min to stain the nuclei. The coverslips were washed with PBS and mounted on glass slides. Fluorescence micrographs were taken using a Zeiss Model Axiovert 200M microscope.

qRT-PCR

RNA was extracted from cells using TRIzol reagent (Invitrogen) according to the manufacturer's instructions. RNA (1 µg) was converted into cDNA using the iScript cDNA synthesis kit (Bio-Rad, Hercules, CA) and used as the template for PCR using Sso Advanced SYBR green Supermix (Bio-Rad) and the CFX96 real-time PCR detection system (Bio-Rad). The quantitative reverse transcription-PCR (qRT-PCR) primers used and their sequences presented in (Table-S1). Threshold cycle (C_T) values were calculated using Bio-Rad CFX manager software. The $2^{-\Delta\Delta CT}$ value was calculated to represent the fold change of the target gene mRNA compared to untreated siRNA control and normalized using β-actin and/or GAPDH as the reference.

Cycloheximide chase assay

Cells were treated with 20 µg/ml cycloheximide (Sigma-Aldrich) for different lengths of time as indicated and the whole cell lysates were prepared. The lysates were subjected

to Western blotting analysis to identify the IL-8 turn-over rate in the si-Tip110 or si-Ctrl-transfected cells. β-actin was used as the loading control.

Analysis of mRNA stability

1205Lu were treated with 20 µg/ml **actinomycin D** (Sigma-Aldrich) to arrest de novo **RNA** synthesis. RNA was extracted using TRIzol reagent (Invitrogen) according to the manufacturer's instructions. qRT-PCR was then performed as previously described with β-actin as the normalization control.

Enzyme-linked immunosorbent assay (ELISA)

IL-8 was measured using a human IL-8 ELISA kit (R&D systems, Minneapolis, MN) according to manufacturer's instructions. The optical density of each well was determined using iMark microplate reader (Bio-Rad) set to the 450 nm with wavelength correction at 595 nm.

Invasions assays

A 24-well BioCoat™ Matrigel® invasion chamber with 8.0 µm PET membrane permeable supports (Ref. 354480, Corning Inc, Bedford, MA) was utilized to assess the invasiveness following the manufacturer's instructions. Chambers were seeded with $2X10^5$ 1205Lu in triplicate and placed in either 10 % FBS containing media which

served as chemo-attractant positive control, 0.1% FBS as a negative control and conditioned media. Following the 16 hr incubation, invaded cells were fixed and stained using Kwik-Diff staining kit (ThermoFisher Scientific, Mississauga, ON) then observed under the microscope and counted in 10 random fields.

Luciferase reporter gene assay

The firefly luciferase activity was determined using the luciferase assay substrate (Promega) according to the manufacturer's instructions. Briefly, cells were washed with ice-cold PBS and lysed with 120 μl 1X firefly luciferase lysis buffer (Promega) at room temperature for 15 min. The lysates were centrifuged at 12,000 ×*g* for 2 min to remove cell debris. The cleared lysates (5 μl) were then mixed with 20 μl firefly luciferase substrate (Promega), and the luciferase activity was measured using an Opticomp luminometer (MGM Instruments, Hamden, CT).

Melanoma patients' survival analysis

Survival analysis was performed using the RNA-seq data published in the TCGA database. The gene expression data were re-normalized using DESeq (PMID: 20979621). All together 469 melanoma patients were processed, but only 455 samples had survival data available. Among those patients, patient ID 9733, 7157 and 3576 were selected for SART3, TP53 and IL-8 expression, respectively. Cox proportional hazard regression analysis was performed and Kaplan-Meier plots were drawn to

visualize the results as described previously (PMID: 23836010). Hazard rate, confidence intervals, and p values were computed using the “survival” R package (https://cran.r-project.org/package=survival).

Data analysis

Statistical analyses were performed by using GraphPad Prism or Excel software. Where appropriate, values are expressed as means ± standard deviations (SD) from triplicate samples. Data were analyzed by the two-tailed Student's *t*-test and two-way analysis of variance (ANOVA) as appropriate. If statistical significance (P <0.05) was determined by ANOVA, the data were further analyzed by Turkey’s post hoc test for multigroup comparisons.

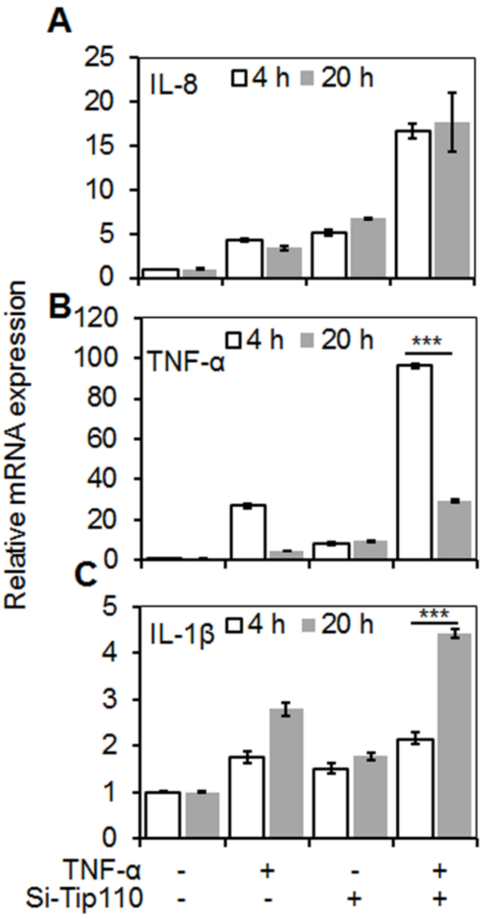

Figure S1. IL-8 induction by Tip110 is TNF-α signaling-independent. A-**C**. 1205Lu were transfected with si-Tip110 or si-Ctrl, cultured for 48 hr, treated with 10 ng/ml of TNF-α for 4 hr or 20 hr, and harvested for total RNA isolation and qRT-PCR to determine the mRNA levels of IL-8 (**A**), TNF-α (**B**), IL-1β (**C**). β-actin was included in the qRT-PCR and used as a relative reference. The mRNA levels in the cells transfected with control siRNA and untreated with TNF-α were set to 1. All the data are representative of triplicate independent experiments and the data presented as the mean ± SE. ***, (*P* < *0.001*); Two-tailed Student's *t*-test.

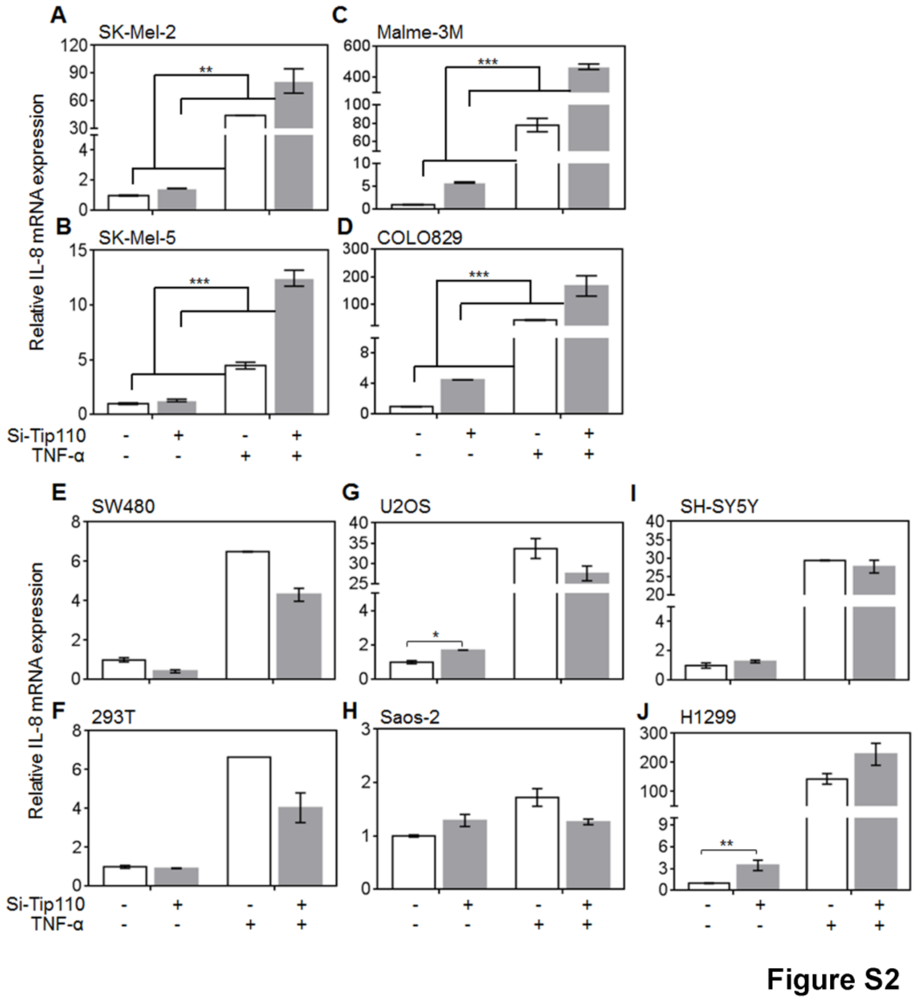

Figure S2. Effect of Tip110 in IL-8 expression in different melanoma cells and cancer types. SK-Mel-2 (**A**)**,** SK-Mel-5 (**B**)**,** Malme-3M (**C**), COLO829 (**D**) Colorectal adenocarcinoma SW480 (**E**), human embryonic kidney fibroblast HEK 293T (**F**), osteosarcoma U2OS (**G**) and Saos-2 (**H**), neuroblastoma SH-SY5Y (**I**), and non-small lung carcinoma H1299 (**J**) were transfected with si-Tip110 or si-Ctrl, cultured for 48 hr, treated with TNF-α (10 ng/ml) for 4 hr, and harvested for total RNA isolation and qRT-PCR to determine the IL-8 mRNA level. β-actin was included in the qRT-PCR and used as a relative reference. The mRNA levels in the cells transfected with control siRNA and untreated with TNF-α were set to 1. All the data are representative of triplicate independent experiments and the data presented as the mean ± SE. * *(P < 0.05)*; ** *(P < 0.01)*; ***, (*P < 0.001*); ANOVA.

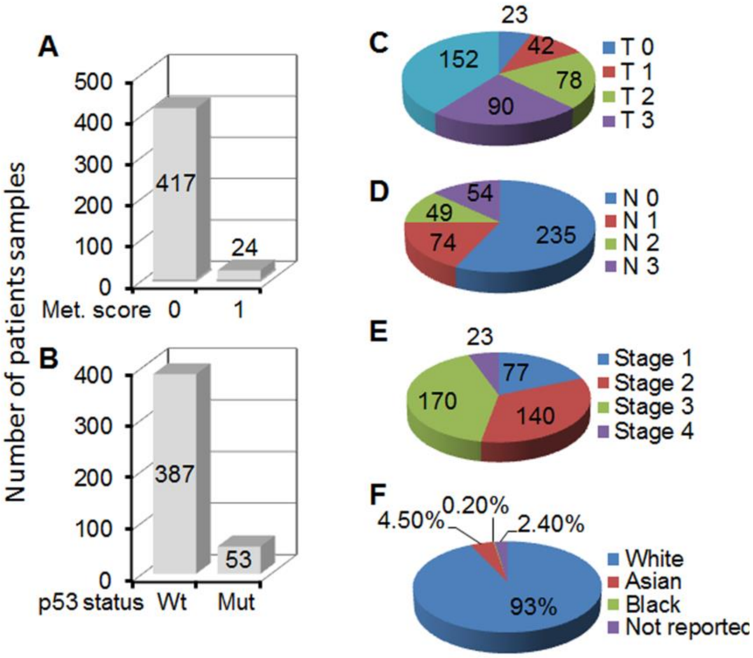

Figure S3

Figure S3. Characteristics of the patients and their tumors. Melanoma patients were categorized based on metastatic (Met) score (**A**), p53 status of the tumors (**B**), primary tumor thickness and how far it has grown within the skin (T) (**C**), metastases to nearby lymph nodes (N) (**D**), the melanoma stages (**E**), and, race (**F**).
