# Supplementary figures and images for: Tip110/SART3 regulates IL-8 expression and predicts the clinical outcomes in melanoma
Source: Mol Cancer. 2018 Aug 17;17:124. doi: 10.1186/s12943-018-0868-z (PMC6098614; doi:10.1186/s12943-018-0868-z)

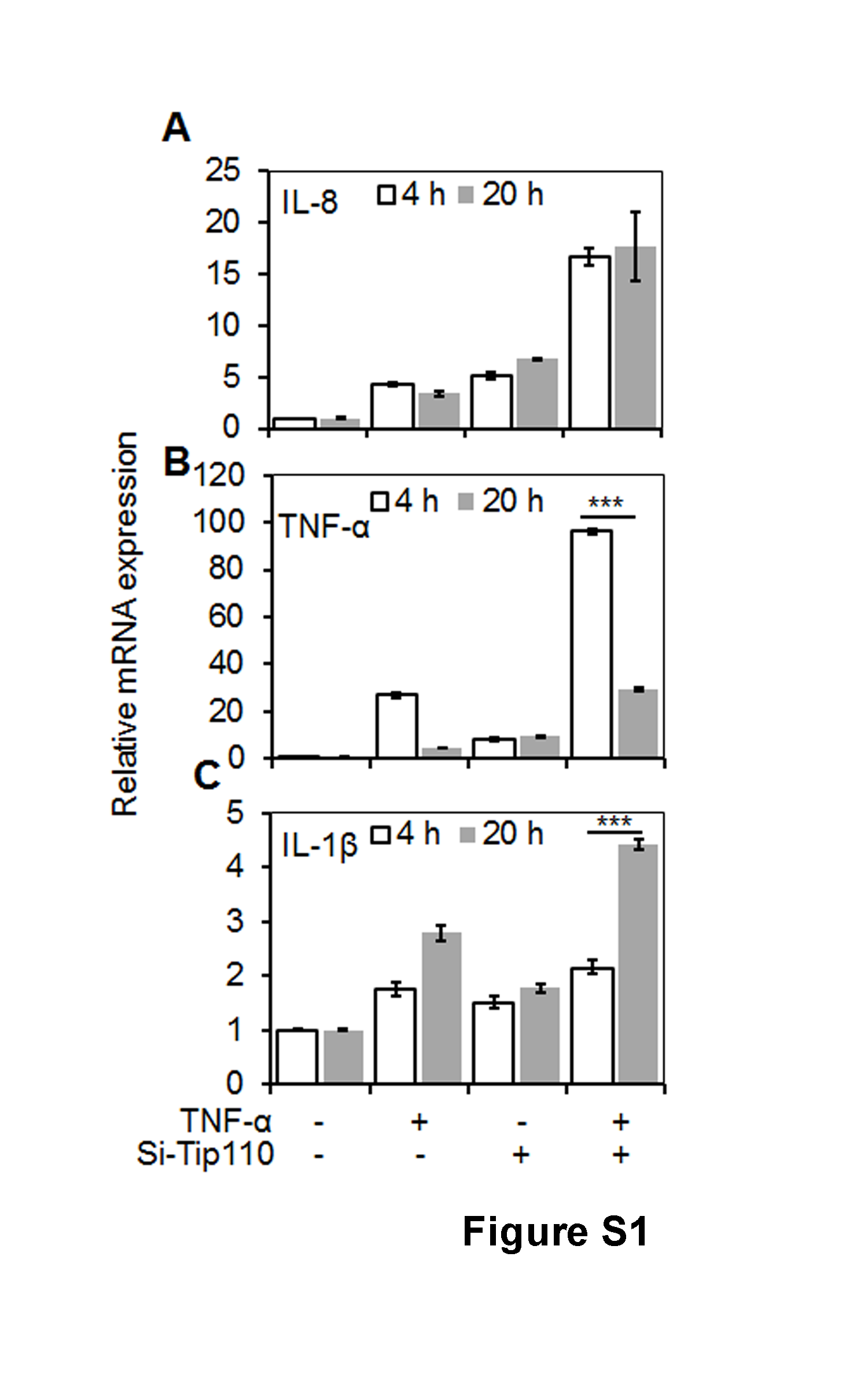

Supplement: Supplementary file 1 — Supplementary materials. (ZIP 3620 kb) [file 12943_2018_868_MOESM1_ESM.zip › Figure S1.tif]

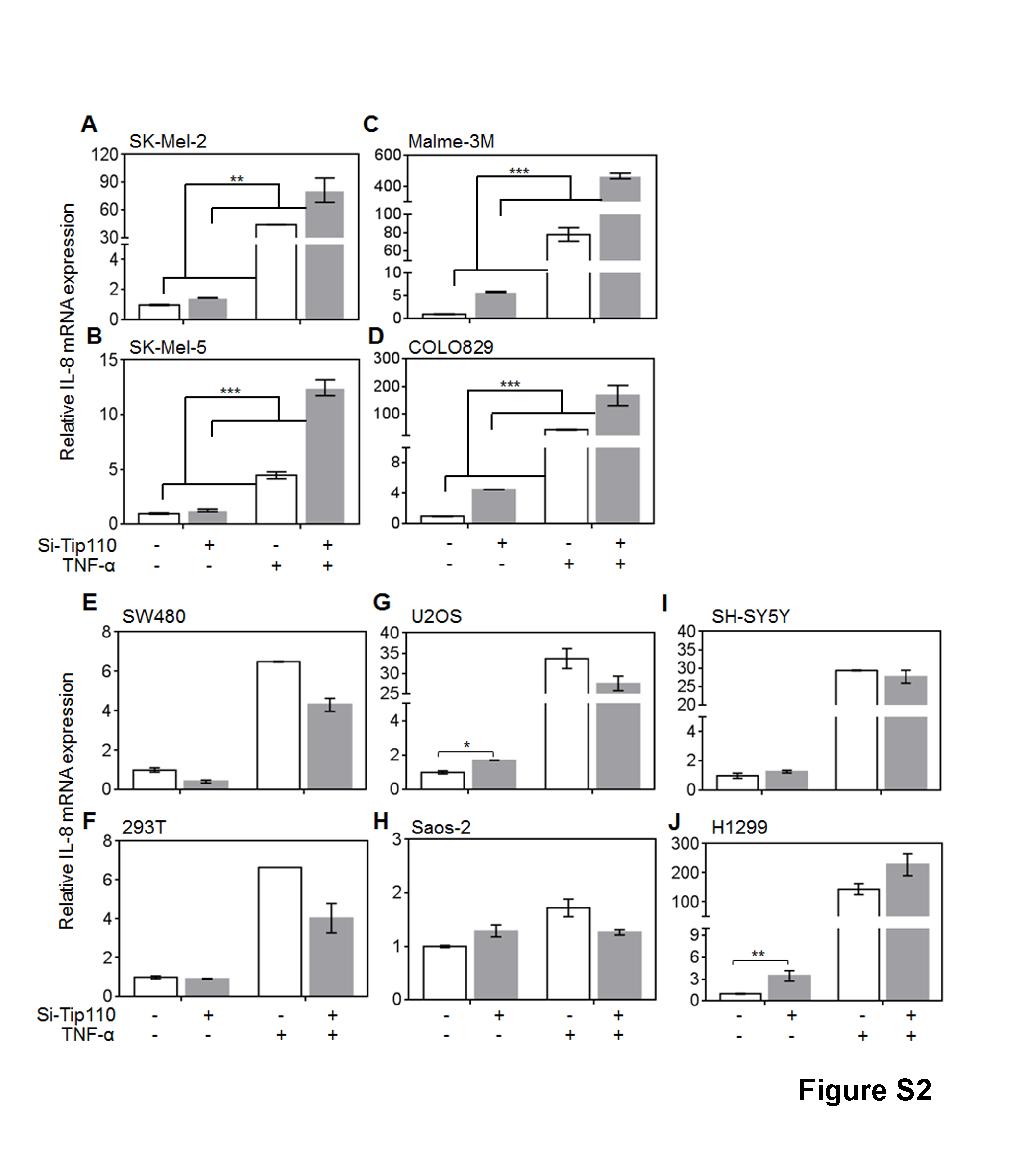

Supplement: Supplementary file 1 — Supplementary materials. (ZIP 3620 kb) [file 12943_2018_868_MOESM1_ESM.zip › Figure S2.tif]

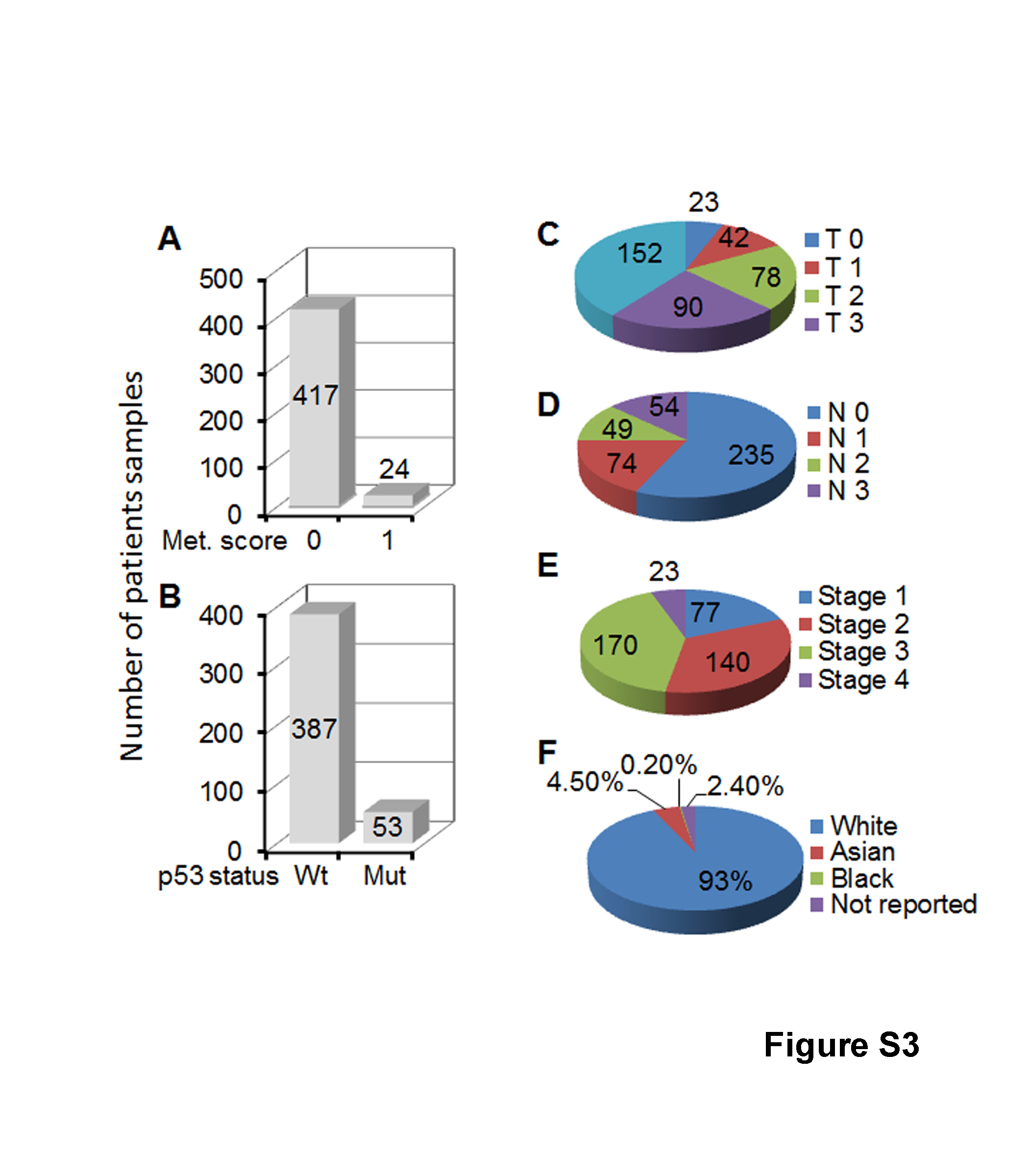

Supplement: Supplementary file 1 — Supplementary materials. (ZIP 3620 kb) [file 12943_2018_868_MOESM1_ESM.zip › Figure S3.tif]
